# Combination Therapies with Kinase Inhibitors for Acute Myeloid Leukemia Treatment

**DOI:** 10.3390/hematolrep15020035

**Published:** 2023-05-24

**Authors:** Shinichiro Takahashi

**Affiliations:** Division of Laboratory Medicine, Faculty of Medicine, Tohoku Medical and Pharmaceutical University, Sendai 983-8536, Japan; shintakahashi@tohoku-mpu.ac.jp; Tel.: +81-22-290-8889

**Keywords:** acute myeloid leukemia, therapy, kinase inhibitors, combination

## Abstract

Targeting kinase activity is considered to be an attractive therapeutic strategy to overcome acute myeloid leukemia (AML) since aberrant activation of the kinase pathway plays a pivotal role in leukemogenesis through abnormal cell proliferation and differentiation block. Although clinical trials for kinase modulators as single agents remain scarce, combination therapies are an area of therapeutic interest. In this review, the author summarizes attractive kinase pathways for therapeutic targets and the combination strategies for these pathways. Specifically, the review focuses on combination therapies targeting the FLT3 pathways, as well as PI3K/AKT/mTOR, CDK and CHK1 pathways. From a literature review, combination therapies with the kinase inhibitors appear more promising than monotherapies with individual agents. Therefore, the development of efficient combination therapies with kinase inhibitors may result in effective therapeutic strategies for AML.

## 1. Introduction

Acute myeloid leukemia (AML) is a clonal disease that is derived from abnormally and, occasionally, poorly differentiated cells of the hematopoietic system [1]. Improvements in AML treatment in younger patients over the past 35 years have largely been due to the dose escalation of chemotherapy and better supportive care [2]. Meanwhile, several attempts to improve antileukemic activity, other than conventional chemotherapy, have emerged. These are epigenetic therapies (e.g., 5-azacitidine), isocitrate dehydrogenase (IDH) inhibitors, and fms-like kinase 3 (FLT3) inhibitors [3,4], which have been employed in clinical practices in these several years. These specific inhibitors were not necessarily developed with the intent of manipulating cellular differentiation; however, they are a central part of the mechanism for these drugs, including cellular differentiation [3].

Accumulated evidences suggests that abnormal activation of signal transduction pathways plays a pivotal role in leukemogenesis, through the blocking of differentiation and abnormal cell proliferation [5]. Kinase inhibitors can serve as differentiation inducers, and the author recently described the application of kinase inhibitors for differentiation therapy in AML [6]. Therefore, targeting kinase activity is considered an attractive therapeutic strategy to overcome AML. Differentiation therapy employing all-trans-retinoic acid for acute promyelocytic leukemia (APL) dramatically improved the clinical outcome in the 1990s. Meanwhile, several attempts to improve antileukemic activity in older patients with diseases other than APL by using hypomethylating agents with low-dose cytarabine showed favorable results [7]. Therefore, the expansion of differentiation therapy for clinical application outside of APL may be attractive for improving clinical outcomes [4]. The author recently described the application of kinase inhibitors for differentiation therapy in AML [6]. Clinical trials for kinase modulators as single agents remain scarce and have shown limited effects [6]. For example, the potential efficacy of epidermal growth factor receptor (EGFR) inhibitors was reported for non-small cell lung cancer, but it was concluded that EGFR inhibitors were not appropriate as single agents for advanced AML [8,9]. Therefore, combination therapy is an area of therapeutic interest that is being pursued. 

To follow this issue in the present review, the author summarizes attractive kinase pathways for therapeutic targets and the combination strategies for these pathways. During a search of the literature on PubMed with the keywords “kinase”, “inhibitor”, “combination”, “AML”, “therapy”, and “clinical”, more than 500 papers were found to have been published to date. Based on the search, the FLT3, PI3K/AKT/mTOR, MAPK, AXL, CDK, and CHK1 pathways appear to be common pathways targeted by kinase inhibitor combination therapies. In the present review, the author summarizes these attractive kinase pathways for therapeutic targets and the combination strategies for these pathways.

## 2. Combination Therapy Targeting the FLT3 Signaling Pathway

Mutations of FLT3 comprise one of the most frequently identified types of genetic alterations in AML [10]. As the biological role of FLT3 is important in the pathogenesis of AML, through the activation of downstream kinase signaling pathways [10,11], the clinical development of FLT3 tyrosine kinase inhibitors (TKIs) has been one of the most active fields in precision medicine for AML [12,13]. 

Several resistance mechanisms for FLT3 inhibitor therapy have been shown, such as additional mutations in the kinase domain in internal tandem duplication (ITD) patients [14], or another kinase pathway activation [15]. To circumvent resistance, as well as to increase efficiency, ongoing efforts are focusing on the development of combinational strategies. We first discovered [16,17], followed by the others [18,19], who showed that FLT3 inhibitor, in combination with arsenic trioxide, acts synergistically on primary AML cells or AML cell lines with FLT3 mutations. Not only for these in vitro models, thus far, reported results of trials combining FLT3 TKIs with induction and consolidation chemo- therapy in the first-line setting have been encouraging [13,20]. Stone et al. [21] previously reported the result of a phase 1b trial. They investigated several schedules and doses of midostaurin, in combination with cytarabine and daunorubicin induction and post-remission therapy of high dose cytarabine in newly diagnosed AML patients. They revealed that midostaurin, in combination with standard chemotherapy, demonstrated high complete response and overall survival (OS) rates in newly diagnosed younger adults with AML. Subsequently, based on the findings of the RATIFY trial, the US Food and Drug Administration (FDA) approved midostaurin in 2017. The FDA approved midostaurin to be used in combination with standard induction therapy with cytarabine and daunorubicin and consolidation therapy with cytarabine in FLT3-mutated newly diagnosed young (18–59 years) patients with AML [22]. Ofran et al. [23] investigated the roles of midostaurin in patients’ survival who were initially treated with intensive chemotherapy plus midostaurin and then proceed to allo-stem-cell transplantation (SCT) in the first complete remission (CR) [23]. In a multivariate analysis, midostaurin use and allo-SCT in CR1 were the most significant factors affecting overall survival (OS). Midostaurin incorporation into chemotherapy regimens significantly improved CR + CR, with incomplete hematologic recovery rates (*p* = 0.002) and reduced relapse rates (*p* = 0.02); it was also remarkably advantageous for FLT3-ITD high-allelic ratio patients (2-year OS of 82%) [23].

FLT3 inhibitors are tyrosine kinase inhibitors and are classified into first- and second-generation inhibitors based on their kinase specificity and potency [24]. First-generation inhibitors include midostaurin and sorafenib. Second-generation inhibitors include quizartinib and gilteritinib. First-generation inhibitors lack specificity to FLT3 and are therefore not as potent as second-generation FLT3 inhibitors, which have been designed to only target FLT3. However, first-generation FLT3 inhibitors can target downstream of FLT3 and may also be effective in parallel signaling and in other targets in AML at diagnosis, which is characterized by the coexistence of multiple leukemic clones [25,26]. Meanwhile, a dominant clone with FLT3 mutations tends to emerge at relapse [26], and it may be better targeted by the second-generation FLT3 inhibitors.

As there are many excellent reviews on FLT3 inhibitors and their combinations [13,27,28,29], in this section, the author introduces essential clinical trials other than FLT3 inhibition plus chemotherapy, such as the combination of the first-generation FLT3 inhibitor sorafenib with the hypomethylating agent 5-azacytidine.

This combination was shown to be well tolerated in older patients with untreated FLT3-ITD AML [30], or underwent a median of 2 prior regimens for treatment (range 0–7) [31]. The majority (53%) of patients experienced grade < 3 adverse effects attributable to sorafenib, and the most common grade ≥ 3 adverse events were thrombocytopenia, neutropenia, anemia, and neutropenia with fever or infection [31]. Another promising combination with FLT3 inhibitor may be venetoclax with decitabine. Maiti et al. [32] reported that the outcomes of newly diagnosed patients with a 2-year OS of 80% compare favorably with prior reports [30] of sorafenib with low-intensity therapy, yielding overall response rates of 78% and a median OS of 5.3–9.2 months.

## 3. Combination Therapy Targeting the PI3K/AKT/mTOR Signaling Pathway

**Figure 1:**
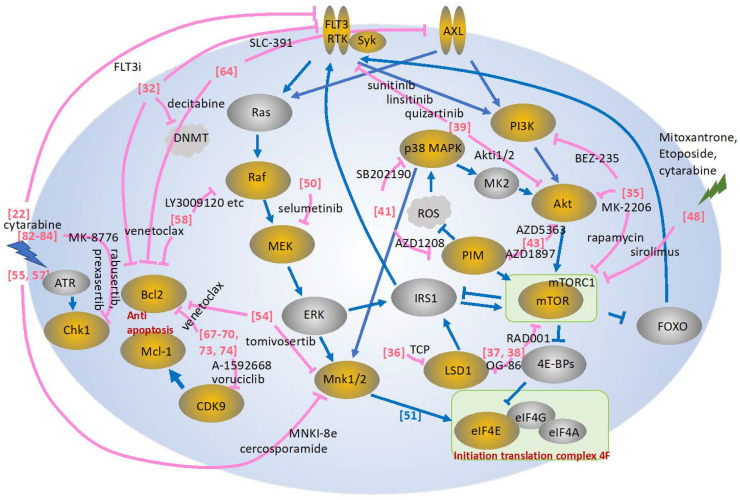
Depicted is a schematic presentation of the pathways described in this review. Effects of inhibitors are shown in pink [22,32,35,36,37,38,39,41,43,48,50,51,54,55,57,58,64,67,68,69,70,73,74,82,83,84]. Yellow circles show the molecules frequently targeted by the kinase inhibitors, while gray circles are less frequently targeted, described in the review. MK2, MAPK-activated protein kinases; IRS1, insulin receptor substrate 1; Mnk1/2, MAPK-interacting kinase 1/2; FOXO, Forkhead box O; mTORC1, mTOR complex 1, TCP: tranylcypromine.

The phosphatidylinositol 3-kinase/AKT/mammalian target of rapamycin (PI3K/AKT/mTOR) signaling pathway is one of the key aberrant intracellular axes in AML [33]. Chemotherapeutic combinations such as cytarabine with AKT inhibitor (MK-2206) have been reported [34]. The PI3K/AKT/mTOR signaling pathway, related pathways, and molecule-specific inhibitor combinations are depicted in Figure 1. Sandhöfer et al. [35] demonstrated a broad range of cytotoxic activities for the inhibition of PI3K/mTORC1/2 (BEZ-235), MK-2206, and TORC1 (rapamycin), with high efficacies for cells carrying a lysine methyltransferase (KMT) 2A rearrangement [35]. The pharmacologic inhibition of lysine-specific demethylase 1 (LSD1) promoted a differentiation blockade especially in AML cells with MLL chromosomal translocations [36]. Deb et al. [37] found that mTORC1 signaling was a target of LSD1. From a dropout screen of a genome-wide CRISPR-Cas9, they revealed multiple components of mTORC1 signaling by LSD1 inhibition (LSDi). They also demonstrated that mTORC1 pharmacologic inhibition with LSDi enhanced differentiation in both the cell line and primary cell settings [37]. Abdel-Aziz et al. [38] found that mTOR was involved in mediating the resistance of leukemic cells to LSDi. Of note, the inhibition of mTOR unlocked the resistance of AML cell lines and primary patient-derived blasts to LSDi both in vitro and in vivo [38].

Bertacchini et al. [39] investigated 80 samples of primary cells from AML patients and found that inhibition of Akt and mTOR resulted in paradoxical activation of growth factor receptor tyrosine kinases (RTKs). Accordingly, dual inhibition of RTKs and AKT displayed synergistic potent cytotoxic effects in a pre-clinical model [39]. However, in a phase II study on 23 AML patients with RAS mutations, combined MEK and AKT inhibition had no clinical activity [40]. This may be explained by the fact that the maximum tolerated clinical dose might not reflect the dosing necessary to produce the desired biological effect of this combination [40].

Another kinase pathway related to AKT/mTOR signaling involves PIM kinases and p38a (MAPK14) [41]. Signaling from Akt and PIM kinase converges to control output from the mTOR signaling axis via regulation of upstream and downstream effectors [42]. PIM kinases (PIM1, 2, and 3) are involved in cell proliferation and survival signaling and are emerging as therapeutic targets for various malignancies. Dual inhibition with PIM kinase and AKT inhibitors was reported to show synergistic cytotoxicity in AML [43]. 

Meja et al. [43] found that a significant portion of primary AML samples showed PIM1 and PIM2 expression, and thus examined the effect of pan-PIM inhibitor AZD1897 on AML cell growth and survival. In their study, PIM inhibition had limited single-agent activity in AML cell lines and primary AML cells, but significant synergy was seen when AZD1897 was combined with Akt inhibitor AZD5363 [43]. PIM kinases are often overexpressed in AML and other hematological malignancies, but the effect of single-agent PIM inhibitor treatment is marginal. Brunen et al. [41] demonstrated that PIM inhibition induced reactive oxygen species production, leading to activation of p38α and downstream AKT/mTOR signaling. Accordingly, inhibition of p38α, combined with PIM kinase inhibition by AZD1208, had a profound effect on AML cells [41].

Recently, metformin, a classic hypoglycemic drug for diabetes, was reported to synergistically sensitize AML cells to Ara-C through mTORC1/P70S6K pathway inhibition [44]. 

However, concerns about combinations with PI3K/AKT inhibitors have been raised. Liang et al. [45] revealed that GLI1 overexpression in AML cells led to increased AKT phosphorylation and decreased Ara-C sensitivity, which was attenuated by GLI1 inhibition. PI3K inhibition profoundly affected GLI1 expression and co-inhibition of GLI1- and PI3K-induced apoptosis of hematopoietic stem/progenitor cells, raising the possibility for serious side effects of this treatment. 

Clinical trials on mTOR inhibitors have been conducted. In the early 2010s, the results of the phase Ib GOELAMS study of the mTOR inhibitor RAD001 [46] and the phase II GIMEMA study (AML-1107) of temsirolimus [47] were published. Overall, these studies revealed that the inhibitors had acceptable toxicity and led to improved outcomes after treatment. The latter study examined the combination of an mTOR inhibitor, temsirolimus, and low dose of clofarabine in older patients with AML as salvage therapy. The overall remission rate (ORR) was 21% (8% complete remission (CR), and 13% CR without full blood count recovery) in 53 evaluable patients. The median disease-free survival was 3.5 months, and the median overall survival was 4 months (9.1 months for responders). In 2018, a clinical trial combining an mTORC1 inhibitor (sirolimus) and MEC (mitoxantrone, etoposide, and cytarabine) was performed in relapsed, refractory, or untreated high-risk AML patients [48]. The ORR among patients with mTORC1 inhibition and baseline target activation during therapy was 71% (12/17), compared with 20% (2/10) in patients without target inhibition. These data provide clinical confirmation that activation of mTORC1 mediates chemotherapy resistance in AML patients.

## 4. Combination Therapy Targeting the MAPK Signaling Pathway

One of the most aberrantly activated oncogenic pathways in AML is the RAS-RAF-MEK-ERK (MAPK) pathway [49]. However, in clinical trials, the targeting of this pathway by MEK inhibitors was not proven to be effective. Jain et al. [50] previously demonstrated the effect of selumetinib, an oral small-molecule inhibitor of MEK, as a modest single antileukemic agent in advanced AML. In parallel, common drug-related toxicities were mild, such as grade 1–2 diarrhea, fatigue, nausea, vomiting, and skin rash. Together with these, a combination with drugs that target other signaling pathways than MEK should be considered in AML.

MAPK-interacting kinases 1 and 2 (MNK1/2) are downstream effectors of this pathway that control the activation of eukaryotic translation factor 4E (eIF4E) [51] (Figure 1). eIF4E was reported to be overexpressed in AML and to play a role in AML pathogenesis [52,53]. Saurez et al. [54] demonstrated an effect of tomivosertib, the highly selective MNK1/2 inhibitor, on AML cells. The inhibition of Mnk was also reported to enhance the apoptotic activity of cytarabine in AML cells [55]. Furthermore, eIF4E inhibition was shown to enhance the effect of FLT3 inhibitors on both internal tandem duplication and tyrosine kinase domain mutants [56]. Altman et al. [57] examined whether cercosporamide, an antifungal agent that acts as a unique Mnk inhibitor, exhibits antileukemic properties. They found that treatment of AML cells with cercosporamide resulted in dose-dependent suppression of eIF4E phosphorylation and that the combination of cercosporamide with cytarabine resulted in enhanced antileukemic responses in a xenograft mouse model in vivo [57]. 

Antiapoptotic Bcl-2 family members are critical for the survival of AML cells. The combination of venetoclax and tomivosertib showed synergistic anti-leukemic responses in AML cells [54]. In a similar context, Tambe et al. [58] reported that pan-RAF inhibition, but not MEK inhibition, caused cell death in 29% of AML samples. Pan-RAF inhibition was not toxic to normal bone marrow cells. Furthermore, pan-RAF inhibition induced apoptosis in AML cells and synergized with BCL2 inhibition [58]. Cremer et al. [59] conducted a genome-scale open-reading-frame resistance screen and identified RAS-MAPK-ERK pathway activation as a major mechanism of resistance to SYK inhibitors. They further demonstrated that an MEK and SYK inhibitor combination was synergistic in vitro and in vivo [59]. Gefitinib, an EGFR inhibitor, was reported to induce differentiation [60] through an off-target effect of Syk family kinase inhibition [61]. Recently, it was reported that phosphorylated EGFR and EGFR ligand were expressed in 19% and 29%, respectively, of blast cells from APL patients, but not in those from healthy controls [62]. The same study further showed that the combination of gefitinib with ATRA and ATO promoted myeloid cell differentiation in ATRA- and ATO-resistant APL cells [62]. 

## 5. Combination Therapy Targeting AXL

AXL, named after the Greek word “anexelekto”, meaning uncontrolled, is a member of the TAM family of receptor tyrosine kinases. AXL potentially drives cell proliferation through effector molecules in the PI3K/AKT/mTOR, RAS/RAF/MEK/ERK, JAK/STAT, and NF-kB signaling pathways [63]. Inhibition of AXL sensitized AML stem/progenitor cells to venetoclax treatment, with strong synergistic effects in vitro and in xenotransplantation models [64]. It was reported that combined treatment with the DNA methyltransferase inhibitor decitabine and histone deacetylase inhibitor vorinostat synergistically inhibited AML cell viability and induced AXL expression [65]. Triple combination treatment with AXL-specific inhibitor BGB324 further increased the sensitivity compared with the decitabine–vorinostat combination treatment [65].

## 6. Combination Therapy Targeting the CDK Signaling Pathway

C Cyclin-dependent kinases (CDKs) 1, 2, 4, and 6 are mainly involved in regulation of the cell cycle, while CDK7, 8, and 9 play roles in regulating transcription to further influence survival and cell proliferation by driving the target gene expressions [66]. Among the CDKs, CDK9 is probably the most attractive target, in combination with other inhibitors or chemotherapy, for hematological malignancies [67,68,69,70,71,72]. Note that Zeidner et al. [72] demonstrated that Alvocidib, a potent and nonselective CDK9 inhibitor, can be safely administrated prior to 7 + 3 (cytarabine + daunorubicin) induction with encouraging clinical activity. There was one dose-limiting toxicity of cytokine release syndrome. The most common grade ≥ 3 nonhematologic toxicities were diarrhea (44%) and tumor lysis syndrome (34%) [72]. 

**Figure 2:**
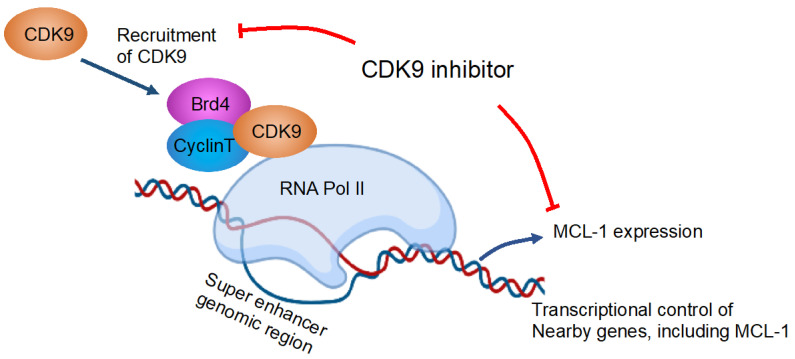
Inhibition of CDK9 reduces MCL-1 expression. The depicted figure is a modification from Tibes et al. [75] that was created with BioRender. Blue arrows and red lines are showing effects and inhibitory effects, respectively. Brd4, bromodomain-containing protein 4; CDK, cyclin-dependent kinase; MCL-1, myeloid leukemia-1; RNA pol II, RNA polymerase II.

CDK9 is a transcriptional regulator of myeloid cell leukemia-1 (MCL-1) that can influence apoptosis induction [73,74]. CDK9 is also a global transcriptional regulator that forms part of the super-elongation complex controlling RNA polymerase II phosphorylation and elongation [73,75] (Figure 2). Several studies have reported effects of other CDKs, such as CDK2 [76] and CDK6 [77,78]. CDK2 suppression was reported to synergize with all-trans-retinoic acid to overcome the myeloid differentiation blockade of AML cells [76].

Recently, histone methyltransferase EZH2 loss and the subsequent reduction in trimethylation of histone H3K27 were reported to result in the de-repression of HOX genes as a novel pathway for acquired resistance to tyrosine kinase inhibitors and cytotoxic drugs in AML [79]. Specifically, CDK1 inhibition prevented the degradation of EZH2, thereby restored drug sensitivity, suggesting the importance of CDK inhibition in leukemia therapy [79].

## 7. Combination Therapy Targeting the CHK1 Signaling Pathway

**Figure 3:**
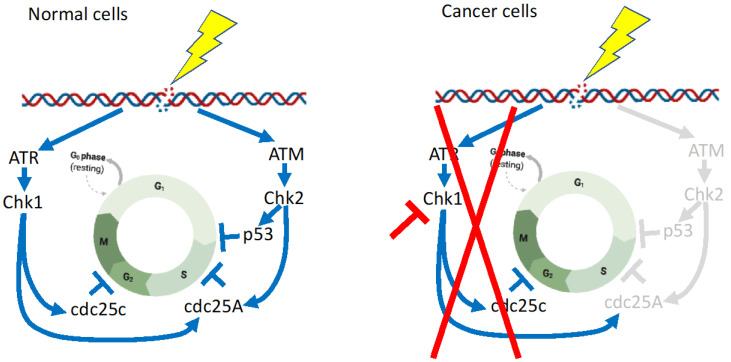
Differences in the DNA damage checkpoint between normal cells and cancer cells. The ATM-CHK2-p53 pathway is impaired in many cancers [80], and thus CHK1 inhibition is very effective for cancers. The depicted figures are modifications from Smith et al. [80] and Goto et al. [81], with minor modifications created with BioRender. Regulatory pathways in normal cells are shown in blue lines and arrows, whereas these are abrogated in cancer cells, shown in red lines. Pathways shown in gray, are inactivated in cancer cells.

The DNA damage checkpoint is regulated by two signaling pathways, ATM-CHK2-p53 and ATR-CHK1-cdc25A, of which the ATM-CHK2-p53 pathway is impaired in many cancers [80] (Figure 3). Consequently, agents that inhibit ATR-CHK1-cdc25A, especially CHK1, are very attractive for the development of efficient therapies [81] (Figure 3). CHK1 is a protein kinase that regulates cell cycle progression in response to checkpoint activation. It was reported that cytarabine, a gold-standard chemotherapeutic agent for AML, activates the replication checkpoint kinases CHK1 and ATR. In turn, cytarabine regulates a series of cellular responses that aid survival during replication stress [82]. Indeed, a selective CHK1 inhibitor was shown to enhance the cytotoxicity of cytarabine [82]. CPX-351 is a liposomal formulation encapsulating cytarabine and daunorubicin that has received approval for treatment of AML. Recently, the addition of a CHK1 inhibitor, MK-8776 or CHK1 knockdown, was found to enhance CPX-351-induced apoptosis in multiple AML cell lines and primary samples [83]. Although there are many promising results in pre-clinical models, clinical success has not been achieved to date [84]. For example, a randomized phase II trial of combination therapy with Ara-C and CHK1 inhibitor MK-8776 produced somewhat disappointing results [85]. Thirty-two patients with relapsed or primary refractory AML were randomized 1:1 to receive either AraC with MK-8776 (Arm A, 14 patients); or AraC alone (Arm B, 18 patients). Response rates and survival were similar between the two groups in spite of evidence that MK-8776 augmented DNA damage in circulating leukemic blasts. There was an increase in asymptomatic grade III prolongation of the QTcF interval among patients receiving MK-8776, Arm B, which was also reported in the phase I trial and is likely attributable to MK-8776 [86]. Di Tullio et al. [87] shed light on another combination strategy involving granulocyte-colony-stimulating factor (G-CSF). They reported that the CHK inhibitor GDC-0575 enhanced the cytotoxicity of Ara-C in different AML cell lines and had effects on AML-cell-line-injected NOD/Scid gamma IL2Rγ null mice. They further revealed that persistent residual leukemic cells became responsive after treatment involving G-CSF administration [87].

A major cause of treatment failure is resistance to chemotherapeutic agents, and one strategy to overcome such chemoresistance is to target the antiapoptotic Bcl-2 protein. The Bcl-2-selective inhibitor ABT-199 showed encouraging preclinical results [88,89]. Mcl-1, a member of the antiapoptotic BCL-1 protein family, is a key regulator of mitochondrial homeostasis [90]. Mcl-1 was demonstrated to contribute to ABT-199 resistance [91]. Zhao et al. [92] found that CHK1 inhibitor LY2603618 decreased Mcl-1. Simultaneous treatment with LY2603618 and ABT-199 resulted in the synergistic induction of apoptosis in both AML cell lines and primary patient samples [92]. 

## 8. Concluding Remarks

Table 1 shows a summary of the agents described in this review. Combinations with FLT3 have been the most attractive form of targeted therapy in AML [30,31,32]. One of the most aberrantly activated oncogenic pathways in AML is the RAS-RAF-MEK-ERK (MAPK) pathway, but in clinical trials, the targeting of this pathway by MEK inhibitors was not proven to be effective [50]. Although there is no clinical activity for MEK and AKT inhibition [40] or Ara-C and CHK1 inhibition [85], several combination therapies have shown clinical efficacy for AML treatment [30,31,32,46,47,48,72]. Among the kinase pathways described in this review (Figure 1), in my opinion, combination therapies that target the FLT3 are the most promising therapies [30,31,32], and also the PI3K/AKT/mTOR signaling pathway shows some efficiency, even in clinical settings [46,47,48]. In addition, at the pre-clinical level, various combination therapies targeting PI3K/AKT/mTOR, MAPK, CDK, and CHK1 pathways seem attractive [35,37,38,41,43,44,67,68,78,82,83]. In the short term, these preclinical and clinical data continue to be rapidly generated—not only FLT3 but others for successful targeted therapy for AML. Determining the optimal combinations and clarifying the mechanisms of inhibitor effects may lead to the development of efficient integrated therapies. The next stage is to decide in which phase of the treatment it should be used, as a part of first-line induction therapy, as consolidation, in a relapse, or in refractory setting. Based on these findings, early recognition of the genomic and prognostic subtype of these gene aberrations, followed by individualized remission–induction or maintenance therapy with kinase inhibitors, is a highly awaited next-generation therapy for AML.

## Figures and Tables

**Table 1:** Combinations of the kinase inhibitors mainly described in this review.

Design of the Study		Results	Refs
Combination Therapy Targeting the FLT3 Signaling Pathway			
Phase III study of whether the addition of midostaurin to standard chemotherapy would prolong overall survival in patients with FLT3 mutation.	Clinical	Overall survival was significantly longer in the midostaurin group than in the placebo group (hazard ratio for death, 0.78; one-sided *p* = 0.009), as was event-free survival (hazard ratio for event or death, 0.78; one-sided *p* = 0.002).	[22]
Phase II study of sorafenib and azacytidine on 27 patients with untreated FLT3 mutated AML	Clinical	The regimen was well tolerated in elderly patients with untreated FLT3 mutated AML with no early deaths.	[30]
Phase II study of sorafenib and azacytidine on 43 AML patients (range, 24–87 years; median, 64 years) were enrolled; 37 were evaluable for response	Clinical	The combination of AZA and sorafenib is effective for patients with relapsed AML and FLT-3-ITD.	[31]
Phase II study of gilteritinib, or sorafenib, or midostaurin, venetoclax and decitabine on 25 patients with FLT3 mutated, newly diagnosed (ND) with AML > 60 years (n = 12) and relapsed/refractory (R/R) patients > 18 years (n = 13).	Clinical	Triplet therapy with FLT3i, venetoclax, and decitabine is safe and an excellent frontline option for older patients with ND *FLT3*mut AML, and it is effective for R/R AML.	[32]
**Combination Therapy Targeting the PI3K/AKT/mTOR Signaling Pathway**			
Specific allosteric AKT inhibitor (MK-2206) and cytarabine in AML cells.	Pre-clinical	MK-2206 is an active agent in AML, and its efficacy in combination with cytarabine is implicated.	[34]
Inhibition of mTORC1 (rapamycin), AKT (MK-2206), and PI3K/mTORC1/2 (BEZ-235) in primary samples and cell lines.	Pre-clinical	Implicating a possible therapeutic benefit in the MLL-mutated subgroup of PI3K/mTOR inhibition.	[35]
mTORC1 pharmacologic inhibition or knockdown of mTORC1 components in combination with LSD1 in both primary cell settings and cell line in vitro and in vivo.	Pre-clinical	Dual LSD1 and mTORC1 inhibition represents a possible combination strategy for enhanced differentiation in AML with MLL-translocation.	[37]
Dual inhibition of Akt and RTKs on AML cells	Pre-clinical	Dual inhibition of Akt and RTKs displays strong synergistic cytotoxic effects in AML cells and downmodulates Akt signaling to a much greater extent than either drug alone.	[39]
Phase II study of combined MEK and AKT inhibition on 23 AML patients with RAS mutations.	Clinical	Combined MEK and AKT inhibition had no clinical activity in patients with RAS-mutated AML.	[40]
p38α inhibitors and PIM kinase inhibitor AZD1208 treatment on hematological tumor cell lines in vitro and in vivo.	Pre-clinical	p38α inhibitors sensitize hematological tumor cell lines to AZD1208 treatment in vitro and in vivo.	[41]
Dual inhibition of PIM and AKT kinase inhibitors in AML cell lines and primary AML cells.	Pre-clinical	A significant synergy was seen when AZD1897 was combined with the Akt inhibitor AZD5363 in AML cell lines and primary AML cells.	[43]
Metformin, Ara-C, and mTORC1/P70S6K pathway inhibition on AML cells.	Pre-clinical	Metformin could synergistically sensitize AML cells to Ara-C via inhibiting the mTORC1/P70S6K pathway.	[44]
Efficacy of RAD001, an mTOR inhibitor, combined with chemotherapy for first-relapsed AML patients.	Clinical	A 70 mg dose of RAD001 at d1 and d7 of an induction chemotherapy regimen for AML has acceptable toxicity and may improve treatment.	[46]
Efficacy of mTOR inhibitor temsirolimus and low dose of clofarabine in older patients as salvage therapy in AML.	Clinical	The predictive value of target inhibition and the acceptable safety profile promote further investigation.	[47]
Sirolimus, an mTORC1 inhibitor, and MEC (mitoxantrone, etoposide, and cytarabine) in high-risk AML patients with untreated, refractory, or relapsed condition.	Clinical	The ORR was 71% (12/17) among patients with mTORC1 inhibition and baseline target activation during treatment, compared with 20% (2/10) in patients without target inhibition.	[48]
**Combination Therapy Targeting the MAPK Signaling Pathway**			
Combination of MNK1/2 inhibitor, tomivosertib, and Bcl-2 inhibitor venetoclax in AML cell lines.	Pre-clinical	Combination of tomivosertib and venetoclax resulted in synergistic anti-leukemic responses in AML cell lines.	[54]
Ara-C with either MNKI-8e, an MNK inhibitor, or knockdown of Mnks by short hairpin RNA in MV4-11 AML cells.	Pre-clinical	In Ara-C-treated MV4-11 cells, the MAPK-Mnk-eIF4E pathway plays a critical role.	[55]
Combination of cercosporamide; Mnk inhibitor, with cytarabine in primitive leukemic progenitors (CFU-L) from AML patients; and a xenograft mouse model.	Pre-clinical	The combination of cercosporamide with cytarabine resulted in enhanced antileukemic responses.	[57]
Pan-RAF inhibitors and BCL2 inhibitor on AML samples and AML cell lines.	Pre-clinical	Pan-RAF inhibition, alone or combined with BCL2 inhibition, is effective in primary AML samples and AML cell lines.	[58]
MEK inhibitor (PD0325901) and SYK inhibitor (entospletinib, PRT062607) in AML cell lines, primary AML samples, and AML model mice.	Pre-clinical	MEK and SYK inhibitor combination was synergistic both in vitro and in vivo.	[59]
**Combination Therapy Targeting AXL**			
Combined treatment with DNA methyltransferase inhibitor decitabine, histone deacetylase inhibitor vorinostat, and AXL-specific inhibitor BGB324 on OCI-AML3 cells and xenograft models.	Pre-clinical	Triple combination increased the sensitivity of OCI-AML3 cells to decitabine and vorinostat, as shown through viability assays, and significantly extended the survival of mice xenograft models.	[65]
**Combination Therapy Targeting the CDK Signaling Pathway**			
CDK9 inhibitor (A-1592668 or the related analog A-1467729) and venetoclax in a number of hematologic cell lines and primary NHL patient samples.	Pre-clinical	CDK9 inhibitor plus venetoclax combination was well tolerated in vivo and demonstrated efficacy superior to either agent alone in both lymphoma and AML mouse models.	[67]
BET bromodomain inhibitor BI 894,999 effect on AML and lymphoma cell line, ex vivo treated AML, and MM primary patient samples and AML xenografts.	Pre-clinical	BI 894,999 is active as monotherapy in AML xenografts and, in addition, leads to strongly enhanced antitumor effects in combination with CDK9 inhibitors.	[68]
CDK inhibitor alvocidib and BCL2 inhibitor venetoclax (ABT-199) on AML cells, AML patient samples, and AML xenograft model.	Pre-clinical	Alvocidib potentiates venetoclax anti-leukemic activity in AML cells, AML patient samples, and AML xenograft models.	[69]
Effect of targeting CDK9 with voruciclib in combination with venetoclax on AML cell lines and primary patient samples.	Pre-clinical	Targeting CDK9 with voruciclib in combination with venetoclax results in synergistic antileukemic activity against AML cell lines and primary patient samples.	[70]
Effect of CDK9 inhibitor, CDKI-73, and BET bromodomain inhibitor JQ1 on AML cell lines and patient-derived xenograft (PDX) model.	Pre-clinical	CDK 9, bromodomain, and extraterminal inhibitors are synergistic in MLL-rearranged leukemia.	[71]
Phase I dose-escalation study of alvocidib on days 1–3, followed by 7 + 3, was performed in newly diagnosed AML ≤ 65 years.	Clinical	Alvocidib can be safely administered prior to 7 + 3 induction with encouraging clinical activity.	[72]
**Combination Therapy Targeting the CHK1 Signaling Pathway**			
Effects of Chk1 inhibitor SCH 900776 and cytarabine were examined using AML cell lines, clinical AML isolates, and normal myeloid progenitors.	Pre-clinical	CHK1 inhibitor SCH 900776 enhanced cytotoxicity of cytarabine in AML lines, clinical AML isolates, and normal myeloid progenitors.	[82]
Effect of Chk1 inhibitor MK-8776 and CPX-351 (a liposomal formulation encapsulating a 5:1 molar ratio of cytarabine and daunorubicin) in AML cell lines and primary AML samples.	Pre-clinical	MK-8776 (CHK1 inhibitor; rabusertib or prexasertib) or CHK1 knockdown enhanced CPX-351 effect and induced apoptosis in multiple AML cell lines and primary samples.	[83]
Randomized phase II trial of Ara-C combined with CHK1 inhibitor MK-8776. Patients with relapsed or primary refractory AML were randomized 1:1 to receive either AraC with MK-8776 (Arm A: 14 patients) or AraC alone (Arm B: 18 patients).	Clinical	Response rates and survival were similar between the two groups.	[85]
Effect of CHK1 inhibitor GDC-0575, Ara-C, and G-CSF in human AML cell line, primary AML cells, human cord blood cells, and AML cell xenografted mice.	Pre-clinical	Combination of CHK1 inhibitor with G-CSF overcame cytarabine resistance in human AML cell lines and had effects on AML-cell-line-injected NOD/Scid gamma IL2Rγ null mice.	[87]
Effect of CHK1 inhibitor LY2603618 and Bcl2 inhibitor ABT-199 in human AML cell line and primary AML cells.	Pre-clinical	Simultaneous treatment with CHK1 inhibitor LY2603618 and ABT-199 resulted in synergistic induction of apoptosis in both AML cell lines and primary patient samples.	[92]

## Data Availability

No new data were created or analyzed in this study. Data sharing is not applicable to this article.
